# Recurrent myocardial injury in a de novo *SON* mutation ZTTK syndrome patient: a case report

**DOI:** 10.1186/s12887-024-04703-4

**Published:** 2024-04-02

**Authors:** Jia Na, Lang Cui, Zhen Zhen, Xi Chen, Qirui Li, Lu Gao, Yue Yuan

**Affiliations:** Department of Cardiology, Beijing Children’s Hospital Capital Medical University, National Center for Children’s Health, Beijing, 100045 China

**Keywords:** ZTTK syndrome, *SON* gene, Myocardial injury, Case report

## Abstract

**Background:**

Zhu-Tokita-Takenouchi-Kim syndrome (ZTTK syndrome) is a severe multi-systemic developmental disorder, caused by variants in the *SON* gene. A patient diagnosed with ZTTK syndrome who carried a de novo *SON* mutation and exhibited recurrent myocardial injury was described in this case.

**Case presentation:**

A 7-year-old girl was admitted to the Cardiology Department of Beijing Children’s Hospital in November 2019 due to myocardial injury following respiratory infection. She displayed elevated myocardial enzymes and severe T-wave changes on electrocardiogram. Over the past three years, she had experienced myocardial injury on three occasions. Additionally, she exhibited intellectual disability, congenital amblyopia, and dysmorphic facial features. Genetic analysis revealed a de novo heterozygous mutation c.3852_3856delGGTAT in the *SON* gene, which was confirmed by Sanger sequencing of her parents. She received anti-infection treatment and was administered metoprolol orally. Her condition was stable at the time of discharge. Over a 42-month follow-up period at the outpatient clinic, she complained intermittent fatigue and palpitation.

**Conclusions:**

The identified *SON* mutation, which plays a crucial role in heart development and mitochondrial function, may be associated with an increased susceptibility to myocardial injury or cardiomyopathy. This case report contributes novel insights into this rare condition and suggests the expansion of the ZTTK syndrome phenotype.

**Supplementary Information:**

The online version contains supplementary material available at 10.1186/s12887-024-04703-4.

## Background

Zhu-Tokita-Takenouchi-Kim syndrome (ZTTK syndrome) is a severe multi-systemic developmental disorder characterized by intellectual disability. It is an autosomal dominant disease caused by variants in the *SON* gene [1]. The syndrome manifests with a range of symptoms, including dysmorphic facial features, musculoskeletal abnormalities, eye abnormalities, digestive system and urogenital system deformities, congenital heart disease, enamel hypoplasia, as well as skin and nail abnormalities [2–4]. The *SON* gene [1], located in the human chromosomal region 21q22.11, consists of 12 exons. It encodes the SON protein, an important RNA splicing co-factor that plays a vital role in the splicing complex [1]. The *SON* gene is also involved in the cell cycle, genome stability, and centrosome maintenance. Animal experiments conducted by Kim et al. demonstrated the essential role of complete *SON* gene in normal growth and development [5]. In 2019, the first case of ZTTK syndrome with a novel heterozygous mutation in China was reported [1]. In this report, we described a patient diagnosed with ZTTK syndrome who carried a de novo *SON* mutation, characterized by recurrent myocardial injury.

## Case presentation

**Fig. 1:**
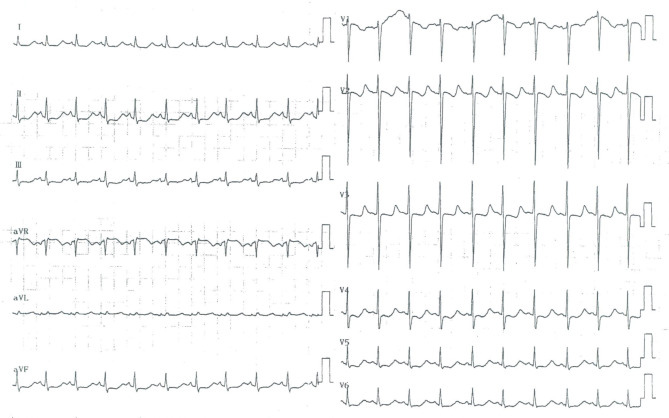
ECG showed widespread ST-T changes on the third admission

**Fig. 2:**
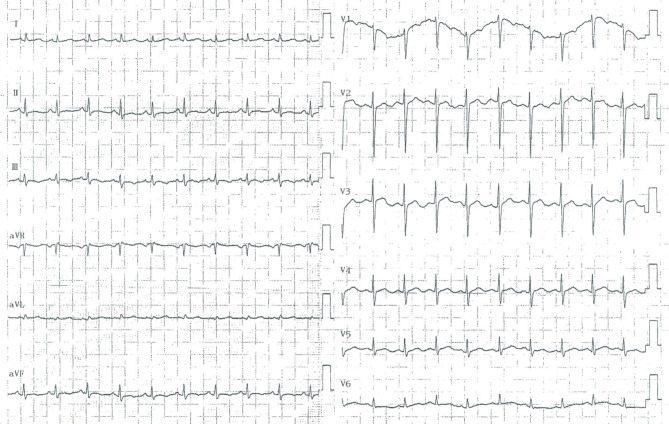
ECG showed improvement upon the third discharge and subsequent reassessment, manifested as partial changes in T waves in certain leads (T-wave flattening in leads II and aVF, and shallow T-wave inversion in lead III)

**Fig. 3:**
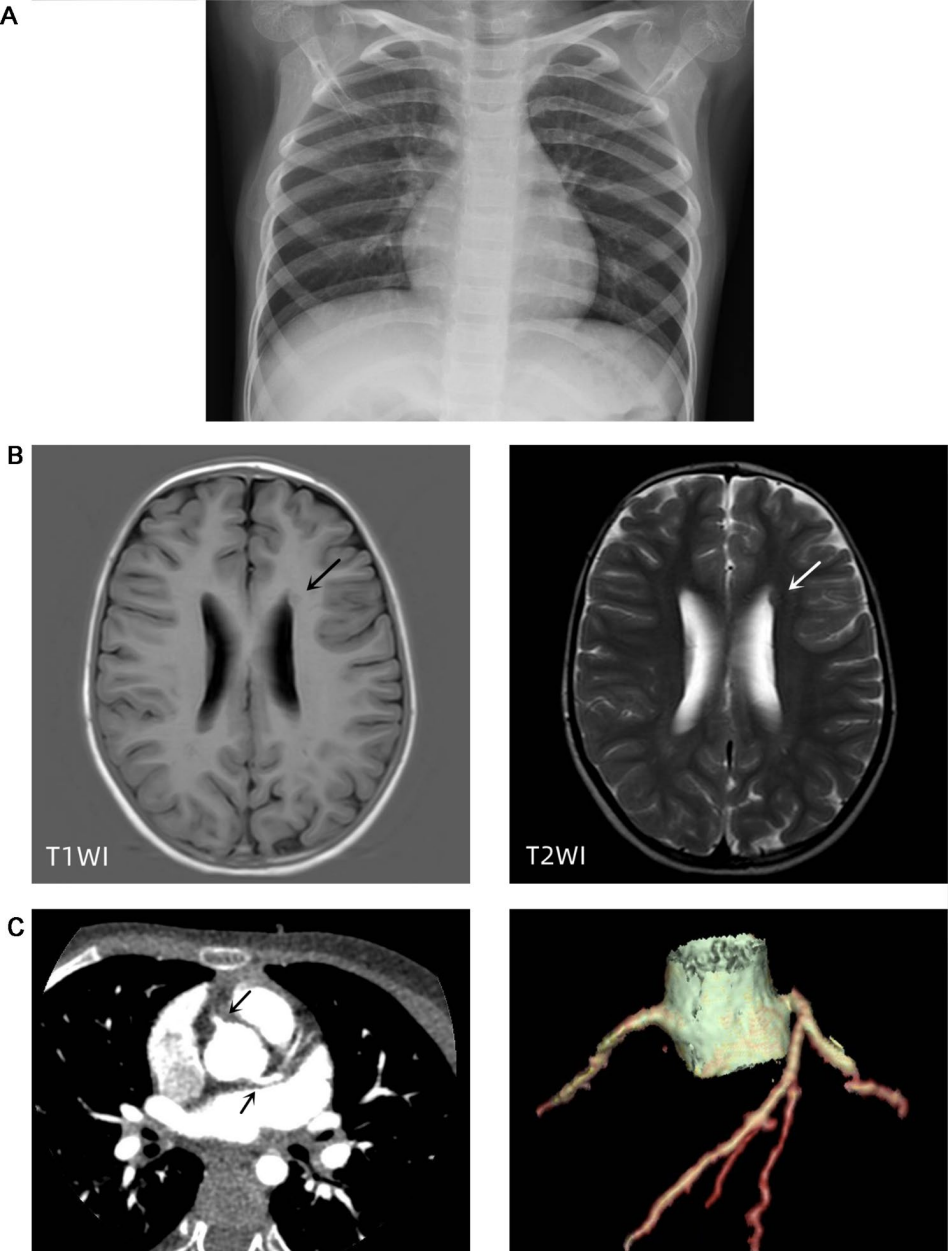
**A** The chest X-ray was normal. **B** Cerebral MRI scan displayed abnormal nodular signals adjacent to the left anterior horn of the lateral ventricle, which the arrows point to. **C** Coronary artery CT angiography showed no abnormality in the origin and course of the bilateral coronary arteries. The arrows point to the location where the left and right coronary arteries originate

**Fig. 4:**
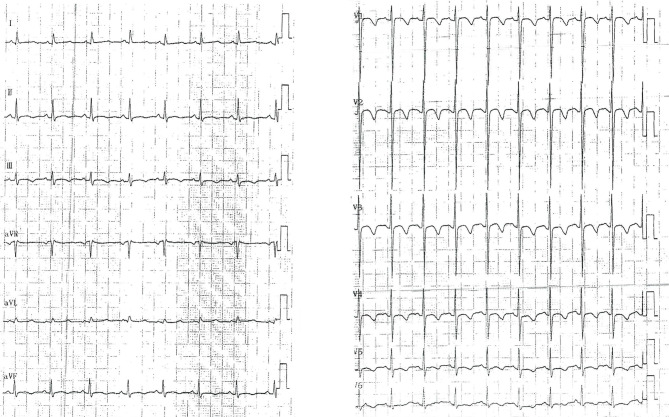
ECG showed severe T-wave changes

A 7-year-old girl presented to the Cardiology Department of Beijing Children’s Hospital with complaints of precordial discomfort and fatigue following a three-day episode of fever and coughing. She had experienced three times of myocardial injury which occurred after acute infection in the previous three years (Table 1). Two previous myocardial injuries were diagnosed as respiratory infection with fever and cough, and one was diagnosed as digestive tract infection with diarrhea. The patient experienced chest pain, chest tightness, and shortness of breath during the course of the disease. Furthermore, these episodes were characterized by elevated myocardial enzyme spectrum indicators and T-wave changes or ST-T changes on electrocardiogram (ECG) (Fig. 1). Between episode, the patient’s electrocardiographic changes showed improvement (Fig. 2). The patient also had intellectual disability and congenital amblyopia. Her parents were healthy and not biologically related. Physical examination revealed that the patient was thin and short, weighing 17 kg and measuring 110 cm in height (both of which are below the 3rd percentile for children of the same age and gender), with distinct facial features such as a narrow mouth, short philtrum, prominent forehead, midface retraction, and mild hypotonia in the limbs. The creatine kinase isoenzyme MB (CK-MB) level was slightly elevated at 51 U/L (reference range: 0–25 U/L). High-sensitivity Troponin-I and N-terminal B-type natriuretic peptide precursor (NT-pro BNP) levels were within normal range. Echocardiogram showed no presence of regional wall motion abnormalities or ballooning of the left ventricle, and no evidence of stress-induced (takotsubo) cardiomyopathy. Abdominal ultrasound revealed multiple gallstones, while chest X-ray showed no abnormality (Fig. 3A). Cerebral magnetic resonance imaging scan displayed abnormal nodular signals adjacent to the left anterior horn of the lateral ventricle, indicating suspected gray matter heterotopia and a cisterna magna cyst (Fig. 3B). Coronary artery computed tomography angiography (CTA) showed no abnormality in the origin or the course of bilateral coronary arteries (Fig. 3C). Twenty-four-hour dynamic ECG revealed an average heart rate of 96 beats/min, with sinus tachycardia accounting for 10% of the total heartbeat. The standard deviation of the NN intervals was 68 ms. The ECG exhibited T-wave changes, specifically T-wave inversion in leads II, III, aVF, V4-V6 (Fig. 4). School-age Wechsler Intelligence Test showed mild impairment which equivalent to 4–5 years old with IQ of 64 points, verbal IQ of 77 points, operational IQ of 57 points, and social adaptation ability of 8 points.

**Table 1:** The disease course of the patient

	Admission time	Duration at admission	Main symptoms	Abnormal laboratory tests	Changes in electrocardiogram	Echocardiogram
First admission	2016.12.21	7 days	Fever, cough, chest pain	CRP 53, CK-MB 31, LDH 340, α- HBDH 312	Sinus tachycardia, low voltage QRS complex in limb leads, T-wave changes	No obvious abnormalities observed
Second admission	2017.7.11	7 days	Diarrhea, chest pain	White blood cells 15.6, LDH 297, α- HBDH 268	Sinus tachycardia, non-specific ST-T changes	No obvious abnormalities observed
Third admission	2018.11.27	3 days	Fever, cough, chest tightness, shortness of breath	CRP 50, CK 679, AST 48.6, LDH 319, α-HBDH 280	Sinus tachycardia, non-specific ST-T changes	No obvious abnormalities observed
Fourth admission	2019.10.26	3 days	Fever, cough, precordial discomfort, fatigue	White blood cells 12.09, CRP 33, CK-MB 51	Sinus tachycardia, T-wave changes	No obvious abnormalities observed

* Reference range for laboratory indicators: White blood cell count (4–10) ×10^9^/L, CRP (C-reactive protein) < 8mg/L, CK-MB (creatine kinase isoenzyme MB) 0–25 U/L, CK (creatine kinase) 25–200 U/L, AST (aspartate aminotransferase) 5–40 U/L, LDH (lactic dehydrogenase) 110–295 U/L, α- HBDH (α-hydroxybutyrate dehydrogenase) 80–220 U/L

**Fig. 5:**
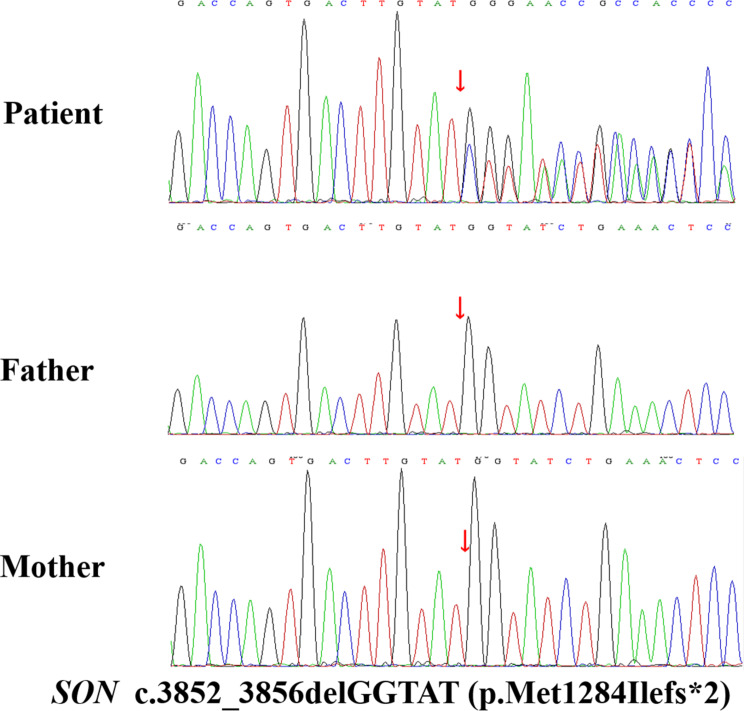
*SON* mutation was confirmed by Sanger sequencing in the patient and her parents. The patient was found to have a heterozygous mutation, c.3852_3856delGGTAT (p.Met1284Ilefs*2), in exon 3 of the *SON* gene (indicated by the red arrow in Fig. 3). This mutation was determined to be de novo, meaning it was not inherited from the patient’s parents

Approximately 2 mL peripheral blood samples of the family members were collected. Whole-exome sequencing (WES, Supplement materials) was performed from the patient’s and her parents’ DNA sample, which identified a heterozygous frameshift mutation, c.3852_3856delGGTAT (p.Met1284Ilefs*2), in exon 3 of the *SON* gene. This mutation was detected in the affected individual but not in her parents, suggesting it was a de novo mutation specific to the patient. Sanger sequencing confirmed this finding in all family members (Fig. 5). The frameshift mutation, c.3852_3856delGGTAT (p.Met1284Ilefs*2), is likely to result in loss of protein function. It is not reported in the 1000 Genome Project, ESP6500 (NHLBI Exome Sequencing Project), ExAC_ALL (Exome Aggregation Consortium), or ExAC_EAS (Exome Aggregation Consortium East Asian) databases. According to the American College of Medical Genetics and Genomics guidelines, the variant was classified into pathogenic, likely pathogenic, uncertain, likely-benign, and benign. Thus, this patient had a pathogenic variant (PVS1 + PS4 + PM2_Supporting + PS2_supporting) [5–10]. This specific mutation has been previously reported in the HGMD by Tokita in 2016 and is known to cause “intellectual disability, congenital malformations, and failure to thrive” [6].

**Fig. 6:**
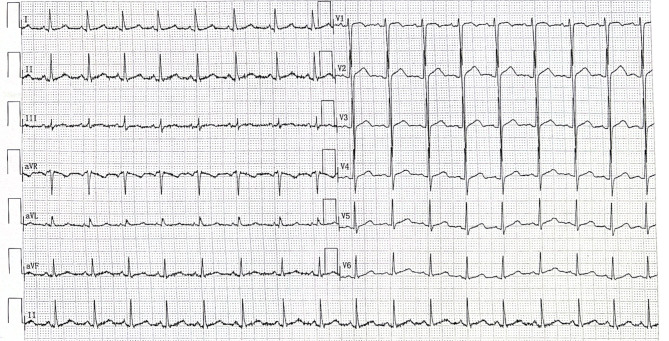
ECG showed improved T-wave changes in May 2023 at the outpatient clinic

Based on the genetic analysis and clinical features, the patient was diagnosed with ZTTK syndrome. A β-blocker (metoprolol) was administrated orally to reduce heart rate as well as myocardial oxygen consumption, combining intravenous with coenzyme Q10. Standard anti-infection treatment was also given. After six days, the patient was discharged with stable body temperature and partially relieved cardiac symptoms. At the age of 8 years and 10 months, the patient visited neurology outpatient department due to learning difficulties, and Binet Intelligence Test scored 89 (marginal). According to the growth curve of children aged between 2 and 18 in China, the height and weight of the patient were below the 3rd percentile for children of the same age and sex, and her BMI, which normally ranging from 18 to 24 kg/m², was below the 25th percentile for children of the same age and sex. During the 42-month follow-up at the outpatient clinic, the patient remained stable, accompanied by intermittent fatigue and palpitation, with a normal echocardiogram and improved T-wave changes on ECG (Fig. 6). She weighed 32 kg at the last follow-up, ranging from the 25th to 50th percentile of children of the same age and sex.

## Discussion and conclusions

In this study, we present a case of ZTTK syndrome in an individual who exhibited recurrent myocardial injury, which is not commonly reported in association with this syndrome. Through next-generation sequencing and Sanger sequencing, we identified a de novo frameshift mutation, c.3852_3856delGGTAT (NM_138927), in exon 3 of the *SON* gene. Exon 3, the largest exon comprising 82% of the coding sequences, was affected by this mutation. The frameshift mutation resulted in a change in the amino acid sequence, premature transcription termination, and complete loss of protein function. This specific mutation has been previously reported in the HGMD by Tokita in 2016 and is known to cause “intellectual disability, congenital malformations, and failure to thrive” [6]. The patient in this study exhibited similar clinical phenotypes to the previously reported case, including low weight and height, distinctive facial features (down slanting palpebral fissures, downturned mouth, short philtrum, thin lip), developmental delay, and hypotonia. However, some clinical phenotypes may vary due to individual expression heterogeneity, environmental factors, and ethnic differences.

Currently, there is no unified diagnostic criterion for myocardial injury in children. In our clinical practice, the diagnosis of myocardial injury in children primarily refers to the “Diagnosis and Treatment of Myocarditis in Children: A Scientific Statement from the American Heart Association” [11, 12]. The diagnostic criteria proposed in this “scientific statement” mainly include a history of preceding infections, cardiac symptoms, abnormal laboratory test indicators, abnormal changes on ECG, and cardiac imaging examinations showing changes in cardiac structure and function. In accordance with the aforementioned diagnostic criteria, in this study: (1) each time the patient fell ill, there was a history of preceding infection, involving respiratory or gastrointestinal infections (manifesting mainly as fever, cough, diarrhea, etc.); (2) the patient exhibited pronounced cardiac symptoms (primarily chest pain, chest tightness, shortness of breath, and fatigue); (3) laboratory test results during hospitalization showed varying degrees of elevation in myocardial enzyme spectrum indicators (including creatine kinase, CK-MB, lactate dehydrogenase, aspartate aminotransferase, α-hydroxybutyrate dehydrogenase), as well as elevated non-specific inflammatory indicators (including peripheral blood leukocyte count, C-reactive protein); (4) the patient’s ECG showed widespread T-wave changes or ST-T changes, which exhibited dynamic variations. All the aforementioned criteria are consistent with the diagnostic criteria in the “scientific statement,” hence the clinical diagnosis of myocardial injury in this case is accurate. Furthermore, following comprehensive treatment including nutritional myocardial support, the patient’s cardiac symptoms were alleviated, and there was improvement in the ECG changes. Indeed, in this case, the elevation of specific serum markers for myocardial injury was not significant, possibly due to the timing of blood sampling, as we might not have obtained blood samples at the peak of indicator abnormalities.

Repeated myocardial injury after infection in this patient is rare in clinical practice. Therefore, after multiple hospitalizations due to myocardial injury or myocarditis, WES test was suggested. The results showed pathogenic variant in the *SON* gene. Due to the rarity of reported cases of ZTTK syndrome, there is currently no report or clear evidence to suggest its association with myocardial injury. A study reported 52 individuals with (possible) pathogenic variations in the *SON* gene [ref]: A broad spectrum of abnormalities was observed, affecting multiple organizational systems with a high clinical inter individual variability. There were significant clinical differences among individuals. And because we did not find any other pathogenic mutations related to the patient’s condition, mutations in the *SON* was considered associated with the susceptibility to myocardial injury or myocarditis. Notably, a significant proportion (31%) of ZTTK syndrome patients have been reported to have heart defects, particularly ventricular and atrial septal defects [2], indicating the importance of the *SON* gene in heart development. *SON* plays an important role in the development of the heart. When the dosage of *SON* haploid is insufficient, the expression of genes involved in embryonic development, neural cell transformation, metabolism, and mitochondrial function is significantly reduced. The mutation in *SON* may affect the mitochondrial function of myocardial cells, thereby affecting their energy metabolism, which may be a pathological mechanism. CTA examination excluded abnormalities in the origin or course of coronary arteries. Multiple normal Troponin-I values and echocardiogram excluded congenital heart disease, indicating non-ischemic myocardial injury. Thus, mutations in *SON* was considered associated with the susceptibility to myocardial injury or myocarditis. However, further research is warranted.

This patient presented with myocardial injury following infection as the primary manifestation and required hospitalization three times over a three-year period. Despite the absence of cardiac structural abnormalities on echocardiogram, elevated myocardial enzymes and changes in ECG indicated myocardial cell damage. Coronary artery CTA revealed no abnormalities in the coronary arteries or main branches, suggesting that the myocardial injury was unlikely to be caused by obstructive coronary arteries. Pathogenic variants in the *SON* gene have been proposed to disrupt mitochondrial function [1, 5]. In this case, we speculated that the recurrent myocardial injury might be associated with abnormal energy metabolism in myocardial cells or abnormal immune response.

In conclusion, a child with recurrent myocardial injury was diagnosed with ZTTK syndrome harboring a heterozygous mutation, c.3852_3856delGGTAT (p.Met1284Ilefs*2), in exon 3 of the *SON* gene. The patient experienced recurrent myocardial injury following infections, suggesting a potential association between the *SON* gene mutation and abnormal myocardial metabolism, as well as an increased susceptibility to myocardial injury. Further investigations, including functional studies, are necessary to establish the pathogenicity of these variants.

### Electronic supplementary material

Below is the link to the electronic supplementary material.

Supplementary Material 1

Supplementary Material 2

## Data Availability

statement. The datasets for this article are not publicly available due to concerns regarding participant/patient anonymity. Requests to access the datasets should be directed to the corresponding author.

## Abbreviations

ZTTK: Zhu-Tokita-Takenouchi-Kim
ECG: electrocardiogram
BNP: B-type natural peptide
CTA: computed tomography angiography
WES: whole-exome sequencing
CK-MB: creatine kinase isoenzyme MB
HGMD: Human Gene Mutation Database
