# Supplementary material for: Recurrent myocardial injury in a de novo SON mutation ZTTK syndrome patient: a case report
Source: BMC Pediatr. 2024 Apr 2;24:232. doi: 10.1186/s12887-024-04703-4 (PMC10985872; doi:10.1186/s12887-024-04703-4)
Supplement: Supplementary file 1 — Supplementary Material 1 [file 12887_2024_4703_MOESM1_ESM.docx]

**NGS Methods**

Written informed consent of the research were provided by the parents. Approximately 2 mL peripheral blood samples of the family members were collected. Whole-exome sequencing (WES) was performed from the patient's and her partents’ DNA sample. Genomic DNA was extracted using QIAamp Blood Midi Kit (QIAGEN, Germany) for DNA library preparation. The amplified DNA was captured by using GenCap Whole-exome capture kit (MyGenostics GenCap Enrichment technologies) and then enrichment libraries were sequenced on Illumina HiSeq X ten sequencer for paired read 150bp. Following quality control, clean reads were mapped to the UCSC hg19 human reference genome using BWA. Duplicated reads were removed using picard tools and mapping reads were used for variation detection. Variants of SNP and InDel were subsequently detected by GATK HaplotypeCaller, using GATK VariantFiltration to filter variant. Variants were further annotated by ANNOVAR and associated with several databases——1000 genome, ESP6500, dbSNP, EXAC, Inhouse (MyGenostics) and HGMD. Select the potential pathogenic mutations as the candidate gene mutations in downstream analysis. On average 95% of the targets had at least 20 reads per base, with an average of 117.14 reads per base.
